# The Role of Molecular Structure in Monte Carlo Simulations of the Secondary Electron Yield and Backscattering Coefficient from Methacrylic Acid

**DOI:** 10.3390/molecules28031126

**Published:** 2023-01-23

**Authors:** Katarzyna Wiciak-Pawłowska, Anna Winiarska, Simone Taioli, Maurizio Dapor, Małgorzata Franz, Jan Franz

**Affiliations:** 1 Faculty of Applied Physics and Mathematics, Gdańsk University of Technology, 80-233 Gdańsk, Poland; 2 European Centre for Theoretical Studies in Nuclear Physics and Related Areas (ECT*-FBK), 38123 Trento, Italy; 3 Trento Institute for Fundamental Physics and Applications (TIFPA-INFN), 38123 Trento, Italy; 4 Advanced Materials Center, Gdańsk University of Technology, 80-233 Gdańsk, Poland

**Keywords:** methacrylic acid, electron–molecule scattering, secondary electron yield, backscattering coefficient, Monte Carlo simulation

## Abstract

In this paper, we show the influence of the chemical structure of four different conformers on the secondary electron emission and backscattering of an electron beam from a gel of methacrylic acid. The conformers have different permanent dipole moments, which determines the cross sections for elastic collisions with electrons. The cross sections are used in Monte Carlo simulations of an electron beam, which enters the gel of methacrylic acid. The secondary electron yield and the backscattering coefficient are computed as a function of the beam energy.

## 1. Introduction

Methacrylic acid (MAA) is a carboxylic acid, which also contains a carbon–carbon double bond. Already in 1880, Engelhorn [1] investigated the polymerization of MAA. Later Trommsdorff [2] suggested MAA as a model compound for proteins. The polymerization reaction can be initiated by light or electrons and is autoaccelerating (Trommsdorff–Norrish effect) [3,4]. The polymerization properties of MAA depend on the structure and orientation of individual MAA molecules [5]. MAA is also an ingredient of the polymer gel MAGIC (Methacrylic and Ascorbic acid in Gelatin Initiated by Copper), which is used for radiation dosimetry by polymerization [6].

Monte Carlo simulations are frequently used to asses the usage of polymer gels for radiation dosimetry (see, e.g., Adinehvand and Rahatabad [7] and Parwaie et al. [8]). In the majority of Monte Carlo simulations, the chemical structure of the individual molecules is not taken into account. In this paper, we show that the chemical structure determines the cross sections for the interaction with electrons and therefore influences the outcome of the Monte Carlo simulations. We found four stable conformers of MAA. In previous studies by Badawi et al. [9] and by Frighetto and Bettega [10], only two of these conformers are discussed.

We present Monte Carlo simulations of secondary and backscattered electrons originated from an electron beam, which enters a gel of MAA molecules. The secondary electron yield and the backscattering coefficient can be measured by elelctron microscopy and are important in the image formation in the electron microscope [11].

Our models for cross sections for elastic collisions, electronic excitation and ionization are based on parameters, which can be obtained from quantum chemical calculations. All cross section models and the Monte Carlo simulation techniques are discussed in Section 2. In addition, we present analytic expressions for the cumulative probabilities, which are derived from the cross sections and are used in the Monte Carlo simulations. In Section 3, we give the details of the quantum chemical calculations, which are used to compute the cross sections. In Section 4, we present the results from the quantum chemical calculations and show the cross sections. Furthermore, we discuss the outcomes of the Monte Carlo simulations. The paper ends with conclusions in Section 5.

## 2. Theoretical Methods

### 2.1. Monte Carlo Simulation Technique

Evaluation of the physical quantities related to the interaction of electrons with MAA was performed by the Monte Carlo simulations based on the energy-straggling strategy (see, e.g., Chapter 6 in [12]), in which all single energy losses occurring along the electron trajectory are taken into account. In this case, the stochastic process for multiple scattering is assumed to follow a Poisson-type law and the numbers $\mu_{i}$ are uniformly distributed random numbers in the range [0,1]. The step-length between two collision processes is thus computed as

(1) $$\Delta s=-\lambda \ln\mu_{\mathrm{step}}\;$$

where the mean free path is given by

(2) $$\lambda =\frac{1}{N\sigma_{\mathrm{tot}}}\;$$

*N* is the number of molecules per unit volume. $\sigma_{\mathrm{tot}}$ is the total cross section. We note that the total cross section is a function of the collision energy and therefore the mean free path is a function of the kinetic energy of the electron. The total cross section is given by the sum

(3) $$\sigma_{\mathrm{tot}}=\sigma_{\mathrm{el}}+\sigma_{\mathrm{inel}}+\sigma_{\mathrm{phonon}}+\sigma_{\mathrm{polaron}}\;$$

Here $\sigma_{\mathrm{el}}$ is the elastic cross section, $\sigma_{\mathrm{inel}}=\sigma_{\mathrm{elect}}+\sigma_{\mathrm{ion}}$ is the sum of the cross sections $\sigma_{\mathrm{elec}}$ for electronic excitation and $\sigma_{\mathrm{ion}}$ for ionization, $\sigma_{\mathrm{phonon}}$ is the electron–phonon cross section, and $\sigma_{\mathrm{polaron}}$ is the cross section for trapping the electron in the interaction site and to create a polaron. The various cross sections are described in more detail in the next subsection.

At the end of each step, it is decided, which kind of process will occur. The cumulative probabilities for this decision process are defined as

$$\begin{array}{ccc} p_{1} & = & \frac{\sigma_{\mathrm{el}}}{\sigma_{\mathrm{total}}}\\ p_{2} & = & p_{1}+\frac{\sigma_{\mathrm{inel}}}{\sigma_{\mathrm{total}}}\\ p_{3} & = & p_{2}+\frac{\sigma_{\mathrm{phonon}}}{\sigma_{\mathrm{total}}}\\ p_{4} & = & 1\; \end{array}$$

A random number $\mu_{\mathrm{type}}$ is generated. Depending on the value of $\mu_{\mathrm{type}}$, it is decided, which type of collision process occurs. For the decision, the following intervals are used.

- If $0\leq \mu_{\mathrm{type}}<p_{1}$, an elastic collision process happens.
- If $p_{1}\leq \mu_{\mathrm{type}}<p_{2}$, an electronic excitation or ionization process happens.
- If $p_{2}\leq \mu_{\mathrm{type}}<p_{3}$, an phonon scattering event happens.
- If $p_{3}\leq \mu_{\mathrm{type}}<1$, the electron induces a polarization and a polaron is created.

The various cases are described in more detail below. Depending on the type of the collision event, the kinetic energy of the electron is reduced. If the kinetic energy is below the value of the electron affinity, the electron is not traced anymore. If an electron is emitted from the surface, its energy is recorded. In the case of an ionization process, a new electron is created at the position of the ionization event and treated by the same algorithm.

The case $0\leq \mu_{\mathrm{type}}<p_{1}$: elastic collision process.

The electron undergoes an elastic collision process, the polar scattering angle θ is determined from differential elastic cross section $\frac{d\sigma_{el}}{d\Omega }\left(E,\theta \right)$. The cumulative probability for this process is defined as

(4) $$P_{\mathrm{el}}\left(\theta ,E\right)=\frac{2\pi }{\sigma_{\mathrm{el}}}\int_{0}^{\theta }\frac{d\sigma_{el}}{d\Omega }\sin\vartheta \;d\vartheta \;$$

Because $P_{\mathrm{el}}\left(\theta ,E\right)$ is a monotonically increasing function in the interval [0,1], we can use a random number $\mu_{\theta }$ to compute the scattering angle θ from the equation

(5) $$\mu_{\theta }=P_{\mathrm{el}}\left(\theta ,E\right)\;$$

The case $p_{1}\leq \mu_{\mathrm{type}}<p_{2}$: electronic excitation or ionization process.

The process is considered an electron excitation event, if the kinetic energy is below the ionization threshold and the electronic excitation energy is substracted from the kinetic energy. Otherwise it is considered an ionization event. Notice that in this work we neglect the resonant emission via Auger and autoionization processes upon ionization and excitation of the molecules. The kinetic energy *W* of the ejected electron is computed with the help of the cumulative probability of the differential ionization cross section

(6) $$P_{\mathrm{ion}}\left(W,E\right)=\frac{1}{\sigma_{\mathrm{ion}}}\int_{0}^{W}\frac{d\sigma_{ion}}{dW^{\prime }}\;dW^{\prime }\;$$

This is a monotonically increasing function in the interval [0,1] and can easily be inverted. With the random number $\mu_{W}$, the energy of the ejected electron is determined as

(7) $$\mu_{W}=P_{\mathrm{ion}}\left(W,E\right)\;$$

After the collision the energy of the primary electron is adjusted by subtracting the binding energy and the kinetic energy of the ejected electron. Furthermore the ionizatin process creates a secondary electron with the kinetic energy *W*, which is propagated with the same algorithm as the primary electron.

The case $p_{2}\leq \mu_{\mathrm{type}}<p_{3}$: electron–phonon scattering.

In that case, a phonon is created by the electron. The phonon energy is substracted from the electron kinetic energy and the deflection angle is determined by (Appendix C in [12])

(8) $$\cos\theta^{\prime }=\frac{E+E^{\prime }}{2\sqrt{EE^{\prime }}}\left(1-B^{\mu_{\mathrm{phonon}}}\right)+B^{\mu_{\mathrm{phonon}}}\;$$

Here, *E* and $E^{\prime }$ are the electron kinetic energy before and after the collision, $\mu_{\mathrm{phonon}}$ is a random number and (Appendix C in [12])

(9) $$B=\frac{E+E^{\prime }+2\sqrt{EE^{\prime }}}{E+E^{\prime }-2\sqrt{EE^{\prime }}}\;$$

The case $p_{3}\leq \mu_{\mathrm{type}}<1$: polaronic effect.

In that case, the electron induces a polarization of the medium around itself creating a polaron. If the random number fullfils the condition, the electron is removed from the simulation.

### 2.2. Models for Cross Sections

#### 2.2.1. Elastic Cross Sections

The collision between an electron and a polar molecule is dominated by the interaction between the charge of the electron and the molecular dipole [13]. We calculate the cross sections using the first Born approximation for the interaction between a charge and a rotating dipole [14]. Because the energy for rotational excitations is in the order of a few meV, the energy loss of the electron is neglected.

The MAA molecule is an asymmetric top molecule, which has three different moments of inertia. The elastic cross section can be written as

(10) $$\sigma_{\mathrm{el}}=\sum_{d}\sigma_{d}^{\mathrm{el}}\;$$

where the sum runs over the three axes of inertia [15]. For each axis of inertia, the partial cross section is given by

(11) $$\sigma_{d}^{\mathrm{el}}=\frac{8\pi }{3k^{2}}D_{d}^{2}\ln\left|\frac{k+k_{d}}{k-k_{d}}\right|\;$$

Here, $D_{d}$ is the component of the molecular dipole moment along the principal axis of inertia with index *d*. The wavenumbers of the electron before and after the collision are given by

(12) $$k=\frac{1}{\hbar }\sqrt{2m_{e}E}\;\mathrm{and}\;k_{d}=\frac{1}{\hbar }\sqrt{2m_{e}\left(E-\hbar \omega_{d}\right)}\;$$

Here, $\hbar \omega_{d}=2B_{d}$ is the energy difference between the first two rotational levels and $B_{d}$ is the rotational constant for the rotation axis with index *d*.

The differential elastic cross section for the collision of an electron with the asymmetric top molecule is given by the sum [15]

(13) $$\frac{d\sigma_{\mathrm{el}}}{d\Omega }=\sum_{d}\frac{d\sigma_{d}^{\mathrm{el}}}{d\Omega }\;$$

In the first Born approximation, the differential elastic cross section along the rotation axis *d* is given by [15]

(14) $$\frac{d\sigma_{d}^{\mathrm{el}}}{d\Omega }=\sum_{\tau^{\prime }}\frac{4D_{d}^{2}}{3}\frac{k_{d}^{\prime }}{k}\frac{1}{\left(k^{2}+k_{d}^{2}-2kk_{d}\cos\theta \right)}\;$$

where θ is the angle between the initial wavevectors and final wavevector of the incident electron. For the determination of the deflection angle of the electron after the elastic collision, the cummulative probability

(15) $$P_{\mathrm{el}}\left(\theta ,E\right)=\frac{2\pi }{\sigma_{\mathrm{el}}}\int_{0}^{\theta }\frac{d\sigma_{\mathrm{el}}}{d\Omega }\sin\vartheta d\vartheta$$

is used. This integral can be solved in closed form and the expression for the cumulative probability is given by

(16) $$P_{\mathrm{el}}\left(\theta ,E\right)=\frac{1}{\sigma_{\mathrm{el}}}\frac{4\pi }{3k^{2}}\sum_{d}D_{d}^{2}\ln\frac{{\left(k+k_{d}\right)}^{2}}{k^{2}+k_{d}^{2}-2kk_{d}\cos\theta }\;$$

#### 2.2.2. Cross Sections for Electronic Excitation

The cross section for electronic excitation is given by [13]

(17) $$\sigma_{\mathrm{elec}}=\sum_{f}\sigma_{f}^{\mathrm{elec}}\;$$

The sum runs over cross sections for electronic excitation from the electronic ground state into the excited states *f*. The integral cross section for each electronically excited state is calculated with the first Born approximation

(18) $$\sigma_{f}^{\mathrm{elec}}=\frac{8\pi }{3k^{2}}D_{0f}^{2}\ln\left|\frac{k+k_{f}}{k-k_{f}}\right|\;$$

Here, $D_{0f}$ is the transition dipole moment between the electronic ground state and the electronically excited state *f* of the molecule. The wavenumbers of the electron before (*k*) and after ($k_{f}$) collision are given by

(19) $$k=\frac{1}{\hbar }\sqrt{2m_{e}E}\;\mathrm{and}\;k_{f}=\frac{1}{\hbar }\sqrt{2m_{e}\left(E-\Delta E_{0f}\right)}\;$$

where $\Delta E_{0f}$ is the electronic excitation energy between the electronic ground state of the molecule and the electronically excited state *f*.

#### 2.2.3. Ionization Cross Sections

The total cross section for direct ionization by electron impact can be written as the sum of the partial ionization cross sections for the $n_{occ}$ occupied orbitals [16,17]

(20) $$\sigma_{\mathrm{ion}}\left(E\right)=\sum_{i}^{n_{occ}}\sigma_{i}^{\mathrm{ion}}\left(E\right)$$

Here, $\sigma_{i}^{\mathrm{ion}}\left(E\right)$ is the partial ionization cross section for ionization from orbital *i*. Within the Binary-Encounter Bethe (BEB) model the partial ionization cross section is given by [16,17]

(21) $$\sigma_{i}^{\mathrm{ion}}=\frac{S_{i}}{t_{i}+u_{i}+1}\left[\left(2-Q_{i}\right)\left(1-\frac{1}{t_{i}}-\frac{\ln t_{i}}{t_{i}+1}\right)+\frac{Q_{i}\ln t_{i}}{2}\left(1-\frac{1}{t_{i}^{2}}\right)\right]$$

Here, $Q_{i}$ is a parameter in the BEB model to take into the account the oscillator strength. If the oscillator strength is unknown (as in our case) these parameters can be set to one. The energy-independent prefactor is given by

(22) $$S_{i}=4\pi a_{0}^{2}N_{i}{\left(\frac{R}{B_{i}}\right)}^{2}$$

where $a_{0}=0.529\times 10^{-10}$ m is the Bohr radius, $N_{i}$ is the occupation number of the orbital and R=13.6 eV is the Rydberg constant. In the above equation, we have used reduced variables

(23) $$t_{i}=\frac{E}{B_{i}}\;\;\mathrm{and}\;\;u_{i}=\frac{U_{i}}{B_{i}}\;$$

where *E* is the kinetic energy of the incoming electron, $B_{i}$ is the electron binding energy in orbital *i* and $U_{i}$ is the expectation value of the kinetic energy of the bound electron in orbital *i*. All energies are given in eV.

The differential ionization cross section is given by

$$\begin{array}{ccc} \frac{d\sigma_{i}^{\mathrm{ion}}}{dw_{i}} & = & \frac{S_{i}}{t_{i}+u_{i}+1}\left[\frac{Q_{i}-2}{t_{i}+1}\left(\frac{1}{w_{i}+1}+\frac{1}{t_{i}-w_{i}}\right)\right.\\ & & +\left(2-Q_{i}\right)\left(\frac{1}{{\left(w_{i}+1\right)}^{2}}+\frac{1}{{\left(t_{i}-w_{i}\right)}^{2}}\right)\\ & & \left.+Q_{i}\ln t_{i}\left(\frac{1}{{\left(w_{i}+1\right)}^{3}}+\frac{1}{{\left(t_{i}-w_{i}\right)}^{3}}\right)\right] \end{array}$$

Here, *W* is the energy of the ejected electron and $w_{i}=\frac{W}{B_{i}}$. The cumulative probability can be obtained by integrating the differential cross section up to a given value of the energy of the ejected electron. The integration can be done analytically and gives the formula

$$\begin{array}{ccc} P_{i}\left(w_{i},E\right) & = & \frac{1}{\sigma_{i}}\frac{S_{i}}{t_{i}+u_{i}+1}\left[\frac{Q_{i}-2}{t_{i}+1}\left(\ln\left|\frac{w_{i}+1}{t_{i}-w_{i}}\right|+\ln t_{i}\right)\right.\\ & + & \left(2-Q_{i}\right)\left(\frac{1}{t_{i}-w_{i}}-\frac{1}{w_{i}+1}+1-\frac{1}{t_{i}}\right)\\ & + & \left.\frac{Q_{i}\ln t_{i}}{2}\left(\frac{1}{{\left(t_{i}-w_{i}\right)}^{2}}-\frac{1}{{\left(w_{i}+1\right)}^{2}}+1-\frac{1}{t_{i}^{2}}\right)\right] \end{array}$$

In our simulation, we use the total ionization cross section in order to compute the probability for an ionization process. For the determination of the energy of the ejected electron, we use the cumulative probability for the ionization from the highest occupied molecular orbital (HOMO). Therefore, the energy of the incoming electron after the ionization process is

(24) $$E^{\prime }=E-W-\epsilon_{\mathrm{HOMO}}\;$$

where $\epsilon_{\mathrm{HOMO}}$ is the binding energy of an electron in the HOMO. A procedure, which goes beyond this approach is the CPA100 model, which is discussed by Bordage et al. [18].

#### 2.2.4. Electron–Phonon Cross Sections

The cross section for electron–phonon interaction is calculated using the Fröhlich theory [19,20]. For a given density *N* of molecules per unit volume, the cross section can be written as (Appendix C in [12])

(25) $$\sigma_{\mathrm{ph}}\left(E\right)=\frac{1}{N}\frac{1}{a_{0}}\left[\frac{n(T)+1}{2}\right]\left[\frac{\epsilon (0)-\epsilon (\infty )}{\epsilon (0)\epsilon (\infty )}\right]\frac{\hbar \omega_{\mathrm{ph}}}{E}\ln\frac{1+\sqrt{1-\frac{\hbar \omega_{\mathrm{ph}}}{E}}}{1-\sqrt{1-\frac{\hbar \omega_{\mathrm{ph}}}{E}}}\;$$

where *E* is the energy of the incident electron and $\hbar \omega_{\mathrm{ph}}$ is the phonon energy of the longitudinal vibrations of the lattice. $a_{0}$ is the Bohr radius, ϵ(0) the static dielectric constant and ϵ(∞) the dielectric constant for high frequency. The average number of phonons n(T) at temperature *T* is given by (Appendix C in [12])

(26) $$n\left(T\right)=(e^{\frac{\hbar \omega_{\mathrm{ph}}}{k_{B}T}}-1{)}^{-1}$$

where $k_{B}$ is the Boltzmann constant.

#### 2.2.5. Electron–Polaron Cross Sections

The cross section for an electron to be trapped due to polaronic effects is calculated with the model by Ganachaud and Mokrani [21]

(27) $$\sigma_{\mathrm{pol}}\left(E\right)=\frac{1}{N}S_{\mathrm{trap}}e^{-\gamma_{\mathrm{trap}}E}\;$$

Here, *N* is the density of molecules per unit volume and *E* is the kinetic energy of the electron, $\gamma_{\mathrm{trap}}$ and $S_{\mathrm{trap}}$ are parameters for the energy domain and the size of the trap.

## 3. Computational Details

All parameters, which are used in the calculations of the elastic and ionization cross sections are calculated with the quantum chemistry computer package Gaussian 16 [22]. The aug-cc-pVTZ basis set has been used in all calculations [23]. The geometries, dipole moments, rotational constants and vibrational frequencies have been computed with the Becke, 3-parameter, Lee-Yang-Parr (B3LYP) exchange–correlation functional [24,25].

For the ionization cross sections, the binding energies of the valence electrons have been calculated with the Outer Valence Green’s Function (OVGF) method [26] using aug-cc-pVTZ basis set [23]. The program package Gaussian 16 [22] has been used for these calculations. Since the OVGF method provides the binding energies only for the outer valence orbitals, the binding energies of all other orbitals are calculated using Koopman’s theorem from the Hartree–Fock orbital energies. The average kinetic energy of the bound electrons before the ionization are computed from the Hartree–Fock orbitals.

The computations of the electronically excited states have been performed with the program package TURBOMOLE [27]. Electronic excitation energies and transition dipole moments have been computed with time-dependent density functional theory [28] using the B3LYP functional [24,25] and the aug-cc-pVTZ basis set [23].

The Monte Carlo simulations are performed with the program SEED. The program is based on the Monte Carlo strategies, which are described in the Chapter 6 of Dapor’s book [12]. The cross sections and cumulative probabilities, which are used by SEED are computed with scripts written in AWK and Python. For each primary beam energy ten million trajectories have been computed.

## 4. Results and Discussion

**Figure 1:**
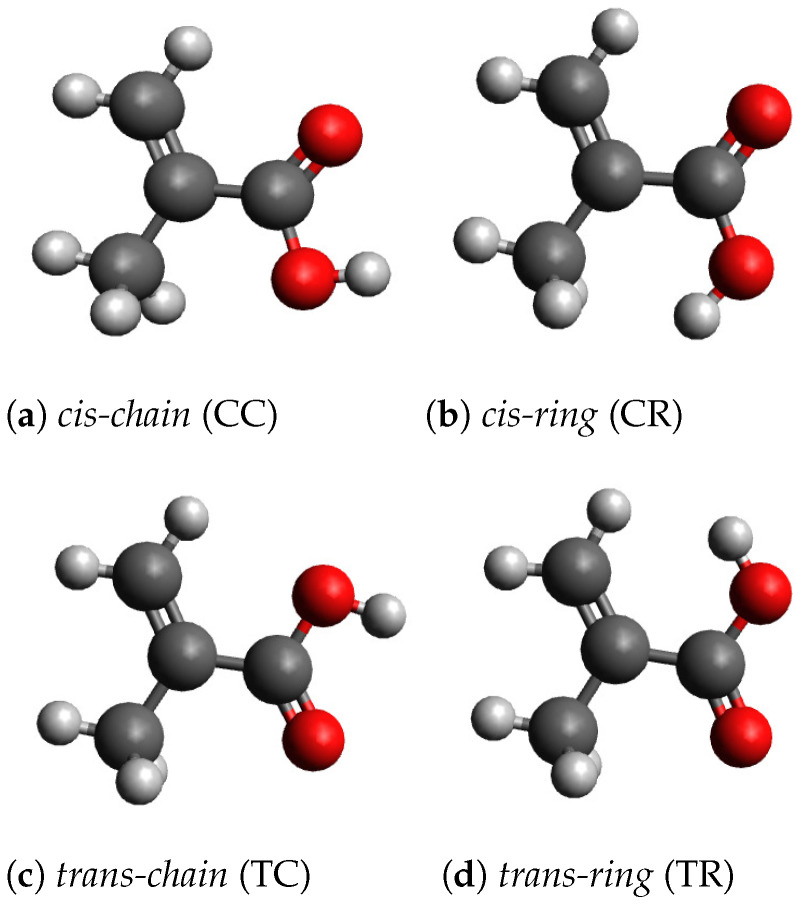
Molecular geometries of the four conformers.

With the geometry optimization, we found four stable conformers, which are shown in Figure 1. For the geometry optimizations, the Berny algorithm [29] is used. For each conformer the vibrational frequencies have been calculated and no imaginary frequencies have been found. In order to distinguish the conformers, we call them *cis*, if the OH-group and the CH${}_{3}$-group are on the same side of two carbons atoms, which connect the two groups. Otherwise the conformers are called *trans*. Furthermore, we distinguish the conformers by the relative orientation of the OH-group. If the Hydrogen atom of the OH-group is rotated to the outside of the molecule, we call the conformer *chain*-like, otherwise *ring*-like. With this naming convention, we can derive four names: *cis-chain* (CC), *cis-ring* (CR), *trans-chain* (TC) and *trans-ring* (TR). The cartesian coordinates of the four conformers are given in Appendix A. The two chain-like conformers CC and TC have been also found by Badawi et al. [9] and by Frighetto et al. [10]. The two ring-like conformers have not been discussed previously. The conformers CC, CR and TC belong to the symmetry point group $C_{s}$. These conformers have one mirror plane, which contains all carbon and oxygen atoms. The geometry of the conformer TR has no mirror plane and the molecule transforms as the point group $C_{1}$. In this case, the COOH-group is rotated out of the plane, which reduces the static interaction between the hydrogen atoms, which carry a small positive charge, in the OH-group and the CH${}_{2}$-group. In the conformer CR, the static interaction between the OH-group and the CH${}_{3}$-group is smaller, because the hydrogen atoms of the CH${}_{3}$-group have larger distances from the hydrogen atom of the OH-group. Table 1 shows the symmetry groups, the total electronic energies $\Delta E_{\mathrm{elec}}$, the enthalpies ΔH and the Gibbs free enthalpies ΔG of the four conformers. TC is the most stable conformer. The conformer CC is about 2.5 kJ/mol less stable. The two *ring*-like conformers, CR and TR, are more than 20 kJ/mol higher in energy. The large energy difference between chain-like and ring-like conformers imply that under equilibrium conditions (at room temperature) less than 1 percent of the molecules will be found in the ring-like conformation. In the condensed phase, the energy difference could be smaller, because the larger dipole moment of the ring-like conformers causes a larger solvation energy. Furthermore, the molecules can be frozen in their conformation.

The most important molecular parameters are collected in Table 2. The dipole moments of the two most stable conformers (TC and CC) are 1.79 D and 1.91 D, respectively. This is in good agreement with the calculations by Badawi et al. [9], who report 1.76 D and 1.88 D using density functional theory (B3LYP) with the 6-311+G** basis set.

The rotational constants for the conformer TC are 5.40, 3.49 and 2.15 GHz, which is in good agreement with the results of the calculations, which have been performed by Badawi et al. [9] (5.374, 3.484 and 2.141 GHz with B3LYP/6-311+G**). For the conformer CC, the rotational constants are 5.33, 3.52 and 2.15 GHz. This is again in good agreement with calculations by Badawi et al. [9] (5.308, 3.517 and 2.143 GHz with B3LYP/6-311+G**).

### 4.1. Cross Sections

**Figure 2:**
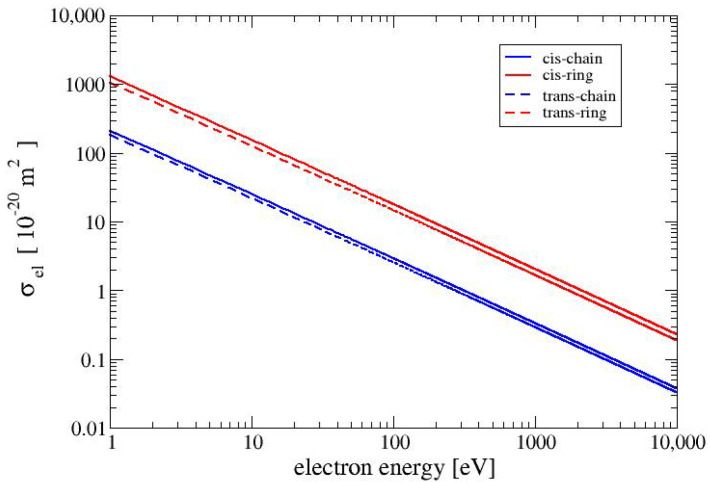
Elastic cross sections for the four conformers.

In Figure 2, we show the elastic cross sections for the four conformers of MAA. For energies above a few eV, the elastic cross sections are proportional to the inverse of the collision energy and show as straight lines in the double-logarithmic plot in Figure 2. The elastic cross sections for the conformer CR is slightly larger than that for the conformer TR. The elastic cross sections for the two ring-like conformers CR and TR are roughly five times larger than those of the chain-like conformers CC and TC. The relative size of the elastic cross sections is due to the square of the absolute values of the molecular dipole moments in Equation (11). The absolute values of the dipole moments of the two ring-like conformers CR and TR are 4.71 D and 4.27 D, respectively. These values are about 2.5 times larger than those of the two chain-like conformers CC and TC (1.91 D and 1.79 D, respectively).

**Figure 3:**
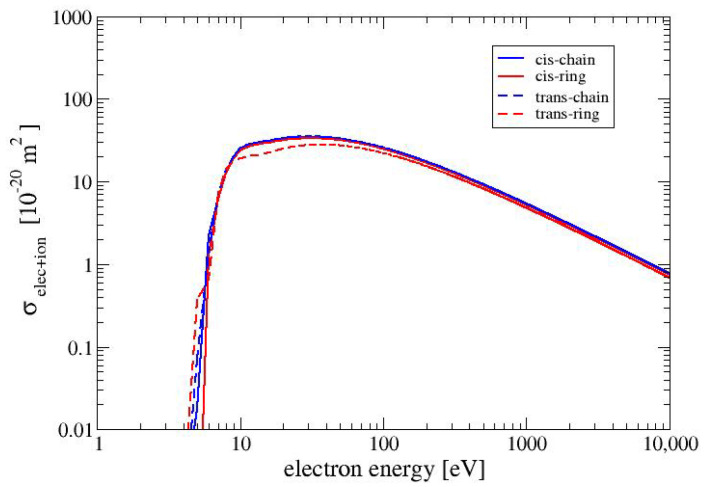
Electronically inelastic cross sections for the four conformers of MAA.

In Figure 3, we show the electronically inelastic cross sections $\left(\sigma_{\mathrm{inel}}=\sigma_{\mathrm{elec}}+\sigma_{\mathrm{ion}}\right)$ for the four conformers. The cross sections for the three conformers CC, CR and TC are nearly identical to each other, whereas the cross section for the conformer TR is slightly lower in the region between 10 and 100 eV. The values of the cross sections for electron–phonon interactions for all MAA conformers are the same and for the examined range of electron energies (1 to 10,000 eV) are in the range between $5\times 10^{-24}\;m^{2}$ and $2\times 10^{-27}\;m^{2}$. When the electron energy increases the cross section for electron-phonon interaction decreases as 1/E. For all conformers of MAA, the values of the cross sections for polaronic effect are the same. and they exhibit the smallest values of among all cross sections which were taken into account in this study. It is worth noticing that the values of the cross section obtained for electron–phonon and electron–polaron interactions are much more smaller than those received for the electronically inelastic cross sections. Therefore, Figure 2 and Figure 3 present only elastic and electronically inelastic cross sections.

### 4.2. Inelastic Mean Free Path

Since there is a general lack of data for electron interactions with MAA, we compare the inelastic mean free path in MAA with the inelastic mean free path in glassy carbon. Glassy carbon (also called glass-like carbon) is an allotrop of carbon with a density of 1.5 g/cm${}^{3}$, which is 35% lower than that for graphite [30]. We define the inelastic mean free path as

(28) $$\lambda_{\mathrm{inel}}=\frac{1}{N\sigma_{\mathrm{inel}}}=\frac{1}{N(\sigma_{\mathrm{elec}}+\sigma_{\mathrm{ion}})}\;$$

**Figure 4:**
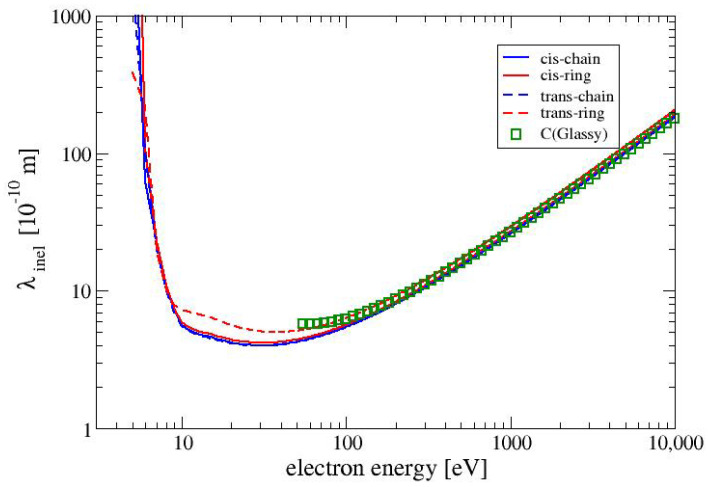
The inelastic mean free path as a function of the electron energy for the four conformers of MAA. Shown also are the values for glassy carbon from calculations by Tanuma et al. [31].

The inelastic mean free paths for all four conformers of MAA are shown in Figure 4 together with the inelastic mean free path for glassy carbon from calculations by Tanuma et al. [31]. As it can be expected from the electronically inelastic cross sections in Figure 3 the mean free inelastic paths of all four conformers are very similar to each other. In the range between 10 and 100 eV the TR conformer shows slightly larger the values of $\lambda_{\mathrm{inel}}$ than the other three conformers. The values of the inelastic mean free path of glassy carbon is very similar for the values the conformer TR.

### 4.3. Spectrum of Emitted Electrons

**Figure 5:**
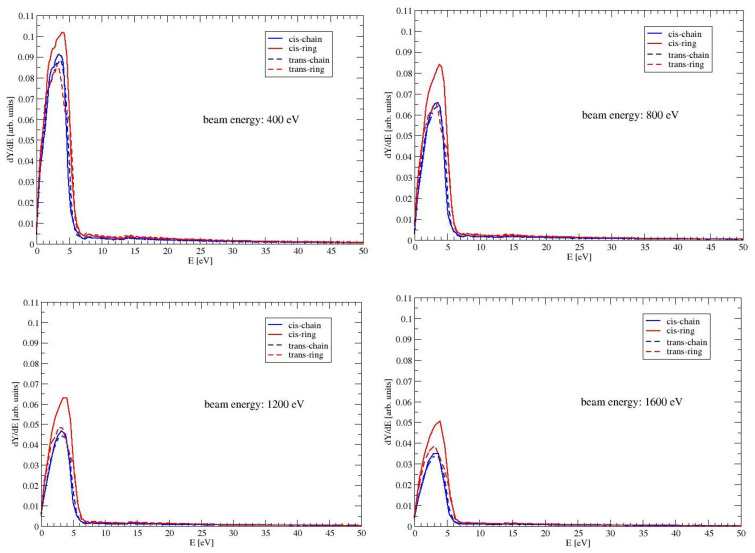
The spectrum of the ejected electrons of the four conformers of MAA. Shown is the low energy part up to 50 eV for beam energies of 400, 800, 1200 and 1600 eV.

In Figure 5, are presented the energy distributions of the electrons, which are emitted from the surface of MAA. The figure shows the spectrum up to 50 eV for beam energies of 400, 800, 1200 and 1600 eV. Most of the electrons are ejected with energies below 6 eV with a maximum between 2 and 4 eV. This can be expected, because MAA has an intensive electronic $\pi \pi^{*}$ excitation at around 6 eV. For all conformers, the energies of the $\pi \pi^{*}$ excitation are listed as $E_{gap}$ in Table 2. If the energy of the electron beam increases, the electrons enter the material with a higher energy. The ionization cross section decreases with increasing energy. Hence the probability for an ionization event in a layer close to the surface decreases. The effect can be seen in Figure 5: the intensity of the peak decreases, when the beam energy increases. The peaks for the conformer CR are higher than those for the other conformers. The difference between CR and the two ring-like conformers is due to the larger elastic cross section of CR. In materials with larger elastic cross sections, the deflection of the incoming electron beam is bigger. Hence the electron beam has a longer path length in a layer close to the surface. Therefore more electrons, that are generated by ionization events, can escape from the material. Even though TR and CR have both large dipole moments, the peaks of TR are visible smaller than those of CR. This is a consequence of the smaller inelastic cross section of TR, which means a lower efficiency in generating ionization events.

### 4.4. Secondary Electron Yield

**Figure 6:**
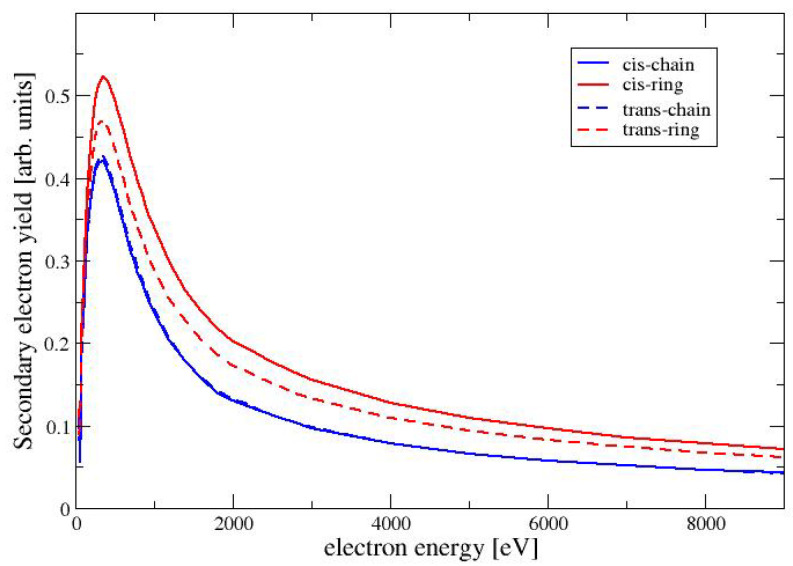
Secondary electron yield as a function of the electron energy for the conformers of MAA.

The secondary electron yield (SEY) for a given beam energy $E_{\mathrm{beam}}$ is defined as integral of the distribution of emitted electrons with energies below 50 eV (see, e.g., Chapter 9 in Dapor [12]). The SEY can be expressed as follows

(29) $$\delta =\int_{0}^{50}\frac{dY}{dE}dE\;$$

where we have used $\frac{dY}{dE}$ as the number of electrons, which are ejected with an energy between *E* and E+dE. In the Monte Carlo code, a discrete value of dE is chosen and the number of ejected electrons in each interval is recorded in a histogram. Routines from the GNU Scientific Library [32] are used for this purpose. It should be noted that we use the expression for the secondary electron yield as a measure for the low-energy electrons, that are emitted from the surface. This should not be confused with the total number of secondary electrons, which are generated by ionization events inside the material and cannot reach the surface. In Figure 6, we show the SEY as a function of the beam energy for the four conformers of MAA. For all conformers, the SEY has its maximum at a beam energy of 350 eV. For higher energies the SEY decreases, because the secondary electrons are generated at a larger depth inside of the material and are trapped within the target. Among the four conformers has the largest SEY, followed by TR and the two chain-like conformers CC and TC. This is the same trend as seen for the elastic cross sections in Figure 2. A larger elastic cross section increases the probability for the deflection of the primary electron beam, also increasing the backscattering of the electrons. The backscattered primary electrons generate secondary electrons before leaving the material. These secondary electons are generated in a layer close enough to the surface to be able to escape. This situation is discussed in more detail in Chapter 3 of the book by Goldstein et al. [11].

### 4.5. Backscattering Coefficient

The backscattering coefficient (BC) for a given beam energy $E_{\mathrm{beam}}$ is defined as the integral of the distribution of emitted electrons with energies above 50 eV (see, e.g., Chapter 8 in Dapor [12]). The BC can be written as

(30) $$\eta =\int_{50}^{\infty }\frac{dY}{dE}dE\;$$

**Figure 7:**
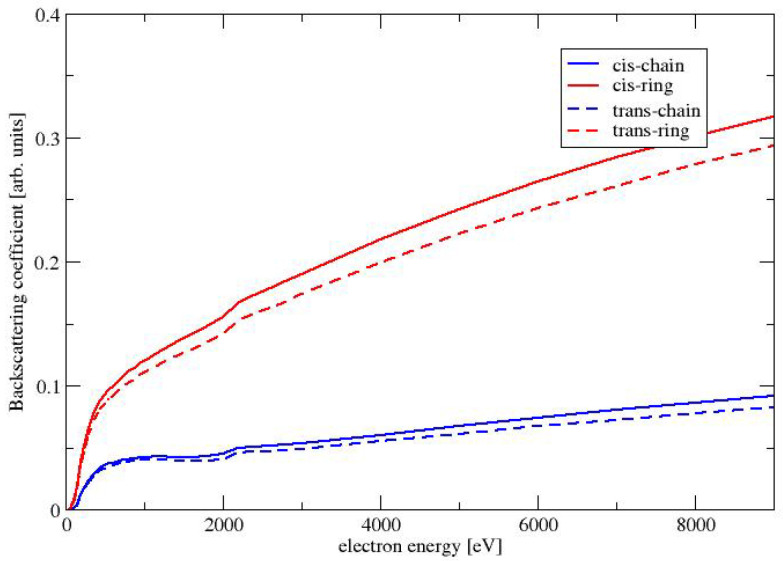
Backscattering coefficient as a function of the electron energy for the conformers of MAA.

The BC for the four conformers of MAA are shown in Figure 7. All BCs are monotonically increasing functions of the energy of the beam energy. As already seen for the elastic cross sections and the SEYs, the BC is largest for the conformers with the largest dipole moment: The largest BC are obtained for the conformer CR, followed by TR. The BCs for the two chain-like conformers CC and TC are much smaller. As already discussed in the previous paragraph about the SEY, a larger elastic cross sections causes a stronger deflection of the primary electrons, which causes a larger BC.

## 5. Conclusions

In this paper, we present the effect of the molecular structure of different conformers of MAA on the cross sections for elastic and electronically inelastic collisions. With Monte Carlo simulations, we investigate the influence of these cross sections on the secondary electron yield and backscattering coefficient of an electron beam, which enters a gel of MAA.

We found four stable conformers of MAA. Previous studies [9,10] found only the two chain-like conformers CC and TC. For the first time, the two ring-like conformers CR and TR are discussed. CR and TR are energetically less stable than the chain-like conformers by about 20 kJ/mol and could be important in the condensed phase. Their dipole moments are about 2 to 3 times larger than those of the chain-like conformers. Therefore the cross sections for elastic collisions are about five times larger for the ring-like conformers than for the chain-like conformers. As a consequence the backscattering coefficient are larger for the ring-like conformers. Due to the larger amount of backscattered electrons a larger amount of secondary electrons is created near the surface and is emitted (see also Chapter 3 in Goldstein [11]).

Our study also shows the influence of the molecular dipole moment, which is usually neglected in Monte Carlo simulations, on the secondary electron yield and the backscattering coefficient.

## Figures and Tables

**Table 1:** Symmetry groups and relative total electronic energies ($\Delta E_{\mathrm{elec}}$), enthalpies (ΔH) and Gibbs free enthalpies (ΔG) of the four conformers of MAA. All values are given in kJ/mol. The values of the most stable conformer (TC) is set to zero. ΔH and ΔG are calculated at a temperature of 298.150 Kelvin and a pressure of one atmosphere.

Conformer	CC	CR	TC	TR
point group	$C_{s}$	$C_{s}$	$C_{s}$	$C_{1}$
$\Delta E_{\mathrm{elec}}$	2.35	25.92	0.0	22.68
ΔH	2.37	25.30	0.0	22.03
ΔG	2.58	25.19	0.0	21.68

**Table 2:** Molecular parameters of the four conformers. Dipole moments (*D*) and its components ($D_{x}$, $D_{y}$ and $D_{z}$) are given in Debye, rotational constants ($B_{x}$, $B_{y}$ and $B_{z}$) in GHz and the ionization threshold ($E_{ion}$), the electron affinity ($E_{EA}$) and the gap energy ($E_{gap}$) in eV.

Conformer	CC	CR	TC	TR
|D|	1.91	4.71	1.79	4.27
$D_{x}$	1.44	2.50	1.35	−2.97
$D_{y}$	−1.25	−4.00	−1.17	2.95
$D_{z}$	0.00	0.00	0.00	0.82
$B_{x}$	5.33	5.28	5.40	5.30
$B_{y}$	3.52	3.51	3.49	3.41
$B_{z}$	2.15	2.14	2.15	2.19
$E_{ion}$	−10.256	−10.454	−10.351	−10.647
$E_{EA}$	0.580	0.389	0.588	0.418
$E_{gap}$	5.83	5.93	5.95	6.08

## Data Availability

The data that support the findings of this study will be soon openly available in the MOST Wiedzy repository (https://mostwiedzy.pl/en/open-research-data/catalog, accessed on 30 October 2022).

## Abbreviations

The following abbreviations are used in this manuscript:

BC	backscattering coefficient
BEB	binary-encounter-Bethe
B3LYP	Becke, 3-parameter, Lee-Yang-Parr density functional
CC	*cis-chain*-like conformer
CR	*cis-ring*-like conformer
D	Debye
DFT	Density Functional Theory
eV	electron volt
GHz	Giga-Hertz
MAA	methacrylic acid
MAGIC	Methacrylic and Ascorbic acid in Gelatin Initiated by Copper
MP2	Møller-Plesset second order perturbation theory
OVGF	Outer Valence Green’s Function
SEY	secondary electron yield
TC	*trans-chain*-like conformer
TR	*trans-ring*-like conformer

## Appendix A

**Table A1:** Optimized geometry of the cis-chain (CC) conformer.

Atom	x	y	z
O	0.9346189	1.7965848	0.0000000
O	−1.3041028	1.9224280	0.0000000
C	−0.3061712	1.2440575	0.0000000
C	−0.2576940	−0.2494828	0.0000000
C	1.0795576	−0.9335940	0.0000000
C	−1.4228608	−0.8948597	0.0000000
H	0.8085916	2.7565113	0.0000000
H	−2.3549987	−0.3483615	0.0000000
H	−1.4656348	−1.9754677	0.0000000
H	0.9541568	−2.0147959	0.0000000
H	1.6672687	−0.6515099	−0.8746298
H	1.6672687	−0.6515099	0.8746298

**Table A2:** Optimized geometry of the cis-ring (CR) conformer.

Atom	x	y	z
O	0.8423816	2.0221396	0.0000000
O	−1.3691634	2.0368648	0.0000000
C	−0.3606584	1.3859107	0.0000000
C	−0.3202111	-0.1193781	0.0000000
C	1.0063430	−0.8315972	0.0000000
C	−1.4864043	−0.7608088	0.0000000
H	1.5715462	1.3925424	0.0000000
H	−2.4143627	−0.2074551	0.0000000
H	−1.5354293	−1.8412617	0.0000000
H	0.8634562	−1.9101224	0.0000000
H	1.6012511	−0.5834171	−0.8838255
H	1.6012511	−0.5834171	0.8838255

**Table A3:** Optimized geometry of the trans-chain (TC) conformer.

Atom	x	y	z
O	1.2346890	1.8396044	0.0000000
O	−1.0073890	1.8718951	0.0000000
C	0.0200500	1.2357640	0.0000000
C	0.0743488	−0.2522614	0.0000000
C	−1.2606060	−0.9377689	0.0000000
C	1.2419804	−0.8947832	0.0000000
H	1.0659909	2.7924234	0.0000000
H	2.1816231	−0.3633705	0.0000000
H	1.2797315	−1.9760832	0.0000000
H	−1.1377877	−2.0189883	0.0000000
H	−1.8463154	−0.6482157	0.8733865
H	−1.8463154	−0.6482157	−0.8733865

**Table A4:** Optimized geometry of the trans-ring (TR) conformer.

Atom	x	y	z
O	1.124721	−1.327637	−0.310174
O	1.688881	0.736496	0.267327
C	−0.678803	0.190585	−0.003865
C	−1.644023	−0.934183	0.225340
C	0.768226	−0.214444	−0.033572
C	−1.055832	1.445961	−0.245443
H	−1.461549	−1.413911	1.188471
H	−2.671848	−0.577986	0.198328
H	−1.509995	−1.704488	−0.534221
H	−2.101791	1.720884	−0.266745
H	−0.353129	2.237733	−0.471946
H	1.252088	1.539374	0.574122
